# Nationwide Hospital-Based Seroprevalence of Hepatitis A and Hepatitis E Virus in Bangladesh

**DOI:** 10.5334/aogh.2574

**Published:** 2020-03-16

**Authors:** Ashraful Islam Khan, M. Salimuzzaman, Md. Taufiqul Islam, Mokibul Hassan Afrad, Tahmina Shirin, Monjur Hossain Khan Jony, Md. Ashraful Alam, Mahmudur Rahman, Meerjady Sabrina Flora, Firdausi Qadri

**Affiliations:** 1 icddr,b (International Centre for Diarrheal Disease Research, Bangladesh), Dhaka, BD; 2 Institute of Epidemiology, Disease Control and Research (IEDCR), Dhaka, BD

## Abstract

**Background:**

Hepatitis A virus (HAV) and hepatitis E virus (HEV) are transmitted by the fecal-oral route and are responsible for epidemic and sporadic outbreaks of acute hepatitis in low-income countries like Bangladesh.

**Objective:**

The purpose of this study was to describe the seroprevalence of acute hepatitis due to HAV and HEV infection in Bangladesh.

**Methods:**

The nationwide food-borne illness surveillance started in 2014 at 10 different hospitals which covered seven divisions of Bangladesh. Blood samples were collected from suspected acute hepatitis cases and screened for the anti-HAV IgM and anti-HEV IgM using enzyme-linked immunosorbent assay (ELISA). Participants’ socioeconomic status, clinical, sanitation and food history were recorded. Multivariate logistic regression was performed to determine the risk factors associated with HAV and HEV infection.

**Findings:**

A total of 998 patients were enrolled and tested for both HAV and HEV. Among these, 19% (191/998) were identified as HAV positive and 10% (103/998) were HEV positive. The median age was 12 years and 25 years for HAV and HEV positive patients, respectively. The prevalence of HAV was higher among the females (24.9%), whereas HEV was higher among males (11.2%). The highest occurrence of HAV was observed among children while HEV was most prevalent in the 15–60 years age group (12.4%).

**Conclusion:**

Through our nationwide surveillance, it is evident that hepatitis A and hepatitis E infection is common in Bangladesh. These data will be useful towards planning preventive and control measures by strengthening the sanitation programs and vaccination strategies in Bangladesh.

## Introduction

Hepatitis A virus (HAV) and hepatitis E virus (HEV) cause acute hepatitis in humans and are transmitted mainly through the fecal-oral route. HAV resulted in approximately 1.4 million cases worldwide annually and 27,731 deaths in 2010, according to the World Health Organization (WHO) [1]. Since the introduction of the hepatitis A vaccine and the start of mass vaccination in several countries in the 1980s, hepatitis A incidence has declined substantially, not only among vaccinated children but in the population as a whole [2,3]. HAV and HEV infections are endemic in many low-income settings. In Asia, many countries have been reported as low, moderate, or high endemic regions for HAV infection [4,5]. Regions of high endemicity include Bangladesh, as well as India, China, Nepal, Pakistan, Myanmar, and the Philippines [6].

HEV is a substantial cause of illness and death world-wide and is responsible for ~20 million infections every year [7]. HEV remains the leading cause of acute hepatitis, and also fatal conditions like acute hepatitis in pregnancy [8] and acute chronic liver failure (ACLF) in Bangladesh [9]. Although persons with HEV disease usually fully recover, clinical studies report that pregnant women who become infected with HEV, and their newborns, often die [10,11,12].

In low-income countries, HAV and HEV are spread by poor food hygiene, unsafe drinking water, and lack of proper sanitation. The risk is higher in rural areas, but a person can be affected anywhere [13]. In Bangladesh, a few studies have been conducted; however, these studies were either restricted to specific areas or related to mortality of pregnant women [14,15,16,17]. The purpose of this study was to describe the seroprevalence of acute hepatitis due to HAV and HEV through a nationwide hospital-based passive surveillance in Bangladesh.

## Materials and methods

### Study area

In May 2014, icddr,b and the Institute of Epidemiology, Disease Control and Research (IEDCR) collaboratively started the diarrheal diseases surveillance system in 10 hospitals. The surveillance was conducted in 10 sentinel surveillance sites (seven district and two tertiary level hospitals, and the Bangladesh Institute of Tropical and Infectious Disease [BITID]) covering all major Divisions of Bangladesh.

### Epidemiological and clinical investigation

The case definition included the discrete symptoms of nausea, anorexia, fever, malaise or abdominal pain, jaundice, and elevated serum aminotransferase levels/serum bilirubin. Trained field attendants and surveillance nurses identified patients (both inpatient and outpatient), according to the case definition from Medicine and Pediatrics Wards. Participants who met the case definition were enrolled after the clinical evaluation by the study physician, five days a week. Upon receiving consent, the physician collected the patient’s socio-demographic characteristics, food history, medical history (including assessment of dehydration status), sanitation and hygiene, and requested a blood sample.

### Specimen collection, storage, transportation and serological test

Five milliliters of blood from adults and 2 ml of blood from children were collected in red-top vacutainers by a trained medical technologist or nurse. For each blood sample, serum was separated, stored at –20°C at the sentinel sites, and later transported to IEDCR twice a week, maintaining the cold chain. HAV IgM and HEV IgM antibodies were detected by Enzyme-linked immunosorbent assay (ELISA) kits (DRG International, Inc., USA), according to manufacturer instructions.

The chi-square (χ^2^) and Fisher’s exact test (if cell frequency < 5) were used to ascertain the statistically significant associations among demographic factors of HAV and HEV infections. The logistic regression model was used to analyze the demographic risk factors. To estimate the crude odds ratio (cOR) with a 95% confidence interval (95% CI), a simple logistic regression model was used to distinguish the probable risk factors between positive and negative hepatitis (HAV and HEV) infections. The adjusted odds ratio (aOR) with 95% CI was calculated using a multiple logistic regression model; the exposure variables considered were age, gender, occupation and education. The two-tailed p-value, p < 0.10 and, p < 0.05 were considered for adjusted model selection and statistical significance, correspondingly. Statistical analyses were performed using Stata 13 software (Stata Corp. 2013. *Stata Statistical Software: Release 13*. College Station, TX: Stata Corp LP). The map was created using statistical programming software R version 3.5.0 (2018-04-23) with ggplot2 packages.

## Results and Discussion

**Figure 1:**
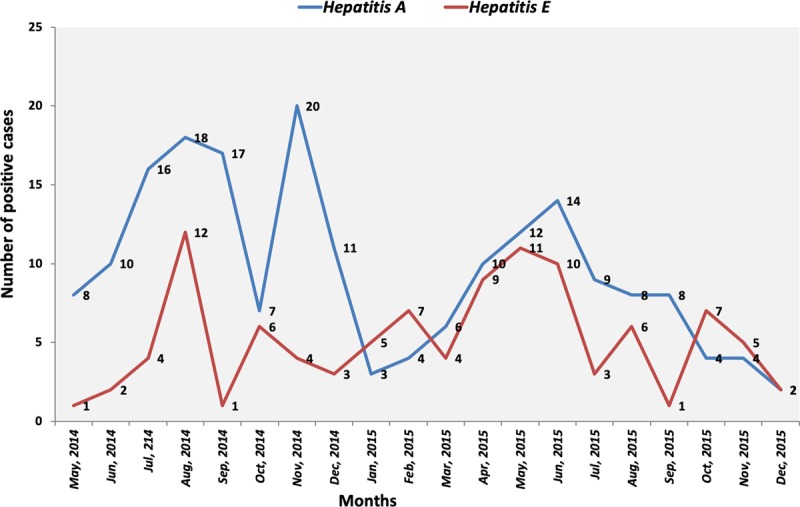
Hepatitis A and Hepatitis E positive patients (in percentage) across the study period.

The study is a comprehensive hospital-based study in Bangladesh demonstrating the burden of enteric acute hepatitis due to HAV and HEV. During the period of May 2014 to December 2015, a total of 998 suspected hepatitis patients were enrolled and screened for the presence of anti-HAV and anti-HEV IgM. Among the tested, 19% were HAV seropositive and 10% were HEV seropositive, resulting in a total of a 29% burden of acute enteric hepatitis (Table 1). The trend of higher HAV seropositivity compared to HEV remained consistent throughout the study period. Susceptibility towards HAV and HEV was observed to be higher from July to September in 2014 and May to July in 2015 (Figure 1). For both infections, positive rates were higher among males (68%); 90% had anorexia, followed by nausea (80%), vomiting (58–60%), fever (75%) and abdominal pain (78%) (Table 2).

**Table 1:** Site-wise distribution of HAV and HEV infection in nationwide surveillance in Bangladesh.

Surveillance Sites	Enrolled patients	Sample tested	HAV, n (%)	HEV, n (%)	Divisions

Narshindi	52	52	11 (21.2)	4 (7.7)	Dhaka
Habiganj	204	204	77 (37.8)	11 (5.4)	Sylhet
Cox’s Bazar	83	83	11 (13.3)	4 (4.8)	Chittagong
Naogaon	70	67	21 (31.3)	20 (29.9)	Rajshahi
Patuakhali	33	33	13 (39.4)	5 (15.2)	Barisal
Thakurgaon	40	40	8 (20.0)	4 (10.0)	Rangpur
Shatkhira	232	232	5 (**2.2**)	8 (**3.5**)	Khulna
DMCH, Dhaka	195	195	14 (7.2)	19 (9.7)	Dhaka
UAMCH, Dhaka	85	73	19 (26.0)	26 (**35.6**)	Dhaka
BITID, Chittagong	22	19	12 (**63.2**)	2 (10.5)	Chittagong
**Total**	**1016**	**998**	**191 (19.1)**	**103 (10.3)**	

**Table 2:** Clinical features of patients (N = 998).

Characteristics	Overall, n (%)	HAV, n (%)	HEV, n (%)

Jaundice	998 (100.0)	191 (100.0)	103 (100.0)
Anorexia	944 (92.9)	165 (86.4)	98 (95.2)
Nausea	814 (80.1)	154 (80.6)	84 (81.6)
Vomiting	585 (57.6)	91 (47.6)	62 (60.2)
Fever	767 (75.5)	154 (80.6)	76 (73.8)
Abdominal pain	797 (78.4)	134 (70.2)	82 (79.6)

**Figure 2:**
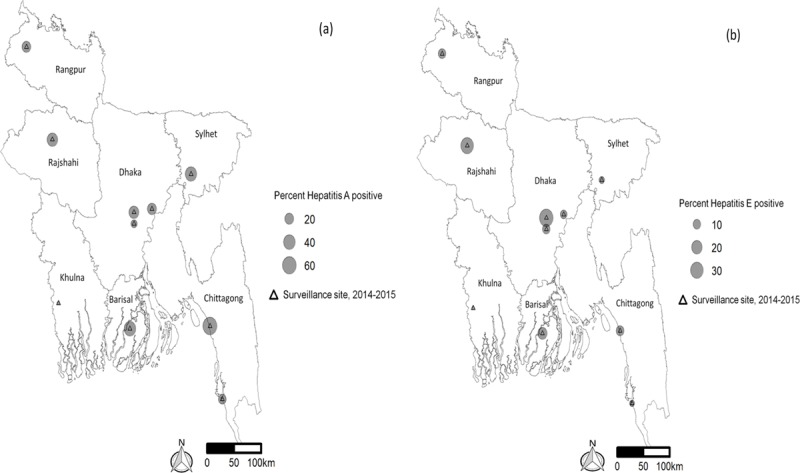
Map of surveillance sites in Bangladesh, showing the percentage of Hepatitis A and Hepatitis E cases among the enrolled febrile illness cases.

Among 998 patients, 191 (19%) were found to be anti-HAV IgM seropositive. However, other studies in Bangladesh show 20–74% with seropositive to anti-HAV among the study population [9,18,19]. Considering the spatial distribution, HAV in Bangladesh exhibited a marked spatial heterogeneity—seropositivity ranged from 2.6 to 40.3%. The highest numbers of HAV seropositive patients were identified in BITID, Chittagong (63.2%), followed by the district hospital in Patuakhali (39.4%). The most affected site identified was BITID, Chittagong (Figure 2a, Table 1). The underlying cause may be attributed to BITID being a well-known infectious disease hospital in that area; patients with complaints of infectious diseases such as hepatitis, febrile illness and diarrheal diseases are generally referred to this hospital. On the other hand, other surveillance sites are general district hospitals or medical college hospitals.

The proportion of HAV was highest among children (<5 years) and young adults (6–14 years) (Table 3) and logistic regression analysis showed that patients 6–14 years of age were 1.6 times more at risk (95% CI = 0.5, 5.2) than the other age categories. The people aged 15–60 years were four times more at risk (95% CI = 1.4, 13.1). Females were 1.8 (95% CI = 1.1, 2.8) times more likely at risk of developing HAV. Amongst different occupations, students were more at risk of HAV, followed by babies/children (~6 times: 95% CI = 1.4, 23.8), businessmen (~3 times: 95% CI = 1.0, 9.5) and service holders (~3 times: 95% CI = 1.1, 9.4), when compared to housewives or the unemployed. Participants who have completed at least secondary school education (SSC) were observed to be less affected by HAV compared to uneducated people (Table 4).

**Table 3:** Demographic characteristics of hepatitis patients in nationwide surveillance in Bangladesh (N = 998).

Characteristics	Samples tested, n (%)	HAV, n (%)	P-value	HEV, n (%)	P-value

**Age**					
0–5	38 (3.8)	26 (13.6)	<0.001	1 (1.0)	0.003
6–14	133 (13.3)	90 (47.1)		5 (4.9)	
15–60	753 (75.5)	73 (38.2)		93 (90.3)	
60+	74 (7.4)	2 (1.1)		4 (3.9)	
**Median age (IQR)**	26 (18-40)	12		25	
**Gender**					
Male	681 (68.2)	112 (58.6)	0.004	76 (73.8)	0.201
Female	317 (31.8)	79 (41.4)		27 (26.2)	
**Occupation**					
Housewife/unemployed	241 (24.2)	12 (6.3)	<0.001	19 (18.5)	0.019
Agricultural work	109 (10.9)	8 (4.2)		10 (9.7)	
Businessman	92 (9.2)	8 (4.2)		10 (9.7)	
Informal worker	127 (12.7)	7 (3.7)		15 (14.6)	
Service	92 (9.2)	7 (3.7)		19 (18.5)	
Students	269 (26.9)	106 (55.5)		27 (26.2)	
Others (Baby/child)	68 (6.8)	43 (22.5)		3 (2.9)	
**Education**					
No formal schooling	130 (13.0)	10 (5.2)	<0.001	7 (6.8)	0.007
Primary and JSC	547 (54.8)	101 (52.9)		58 (56.3)	
SSC and above	251 (25.2)	30 (15.7)		36 (35.0)	
Others (child, not completed primary level)	70 (7.0)	50 (26.2)		2 (1.9)	
**Total**	**998 (100.0)**	191 (100.0)		103 (100.0)	

**Table 4:** Multivariate logistic regression analysis to find out the association of HAV and HEV infection.

Characteristics	Hepatitis A		Hepatitis E	

	cOR^1^ (95% CI)	aOR^1^ (95% CI)	cOR^1^ (95% CI)	aOR^1^ (95% CI)

**Age (year)**				
0–5	1.00	1.00	0.7 (0.1, 6.1)	1.1 (0.1, 18.6)
6–14	1.0 (0.4, 2.1)	1.6 (0.5, 5.2)	1.00	1.00
15–60	0.1*(0.0, 0.1)	0.3 (0.1, 1.2)	3.6*(1.4, 9.0)	4.3*(1.4, 13.1)
60+	0.0*(0.0, 0.1)	0.1*(0.2, 0.9)	1.5 (0.4, 5.6)	2.9 (0.6, 13.6)
**Gender**				
Male	1.00	1.00	1.00	–
Female	1.69*(1.2, 2.3)	1.8*(1.1, 2.8)	0.7 (0.5, 1.2)	–
**Occupation**				
Housewife/unemployed	1.00	1.00	1.00	1.00
Agricultural work	1.5 (0.6, 3.8)	2.4 (0.9, 6.6)	1.2 (0.5, 2.6)	1.1 (0.4, 3.0)
Businessman	1.8 (0.7, 4.6)	3.3*(1.1, 9.4)	1.4 (0.6, 3.2)	1.3 (0.5, 3.4)
Informal worker	1.1 (0.4, 2.9)	1.3 (0.4, 3.8)	1.6 (0.8, 3.2)	1.7 (0.7, 4.2)
Service	1.6 (0.6, 4.1)	3.2*(1.0, 9.5)	3.0*(1.5, 6.1)	2.8*(1.1, 7.1)
Students	12.4*(6.6, 23.3)	8.9*(3.7, 21.5)	1.3 (0.7, 2.4)	1.8 (0.8, 4.1)
Others (Baby/child)	32.8*(15.3, 70.3)	5.8*(1.4, 23.8)	0.5 (0.2, 1.9)	1.7 (0.3, 10.2)
**Education**				
No formal schooling	1.00	1.00	1.00	1.00
Primary and JSC	2.7*(1.4, 5.4)	0.6 (0.2, 1.3)	2.1 (0.9, 4.7)	2.1 (0.9, 4.9)
SSC and above	1.6 (0.8, 3.4)	0.4*(0.1, 1.0)	2.9*(1.3, 6.8)	1.9 (0.7, 5.0)
Others (child, not completed primary level)	30.0*(13.1, 68.6)	1.7 (0.4, 6.9)	0.5 (0.1, 2.6)	1.3 (0.1, 14.2)

^1^ aOR, adjusted odds ratio; cOR, crude odds ratio.

The highest proportion of HEV positive patients (35.6%) were identified in Uttara Adhunik Medical College Hospital which is in Dhaka Division (Figure 2b). The rates were also high in the district hospital in Naogaon in Rajshahi Division (29.9%) (Table 1). A previous study conducted among the apparently healthy population in Bangladesh has shown a 7.3% prevalence of anti-HEV IgM [20]. Another study from Pakistan reported a 17.5% prevalence of IgG to HEV in their population [21].

In contrast to HAV, the proportion of HEV was highest among older adults (15–60 years) and low among children and young adults. Males were predominantly affected by HEV compared to females (Table 3). The findings are similar to other previous reports from Bangladesh [9,15,22,23,24]. Service holders were at least three times more at risk (95% CI = 1.1, 7.1) of developing HEV (Table 4).

The study is the first nationwide surveillance data on HAV and HEV prevalence in Bangladesh. The strength of this surveillance was the collection of data considering a wide geographical and demographic distribution, with large sample size, covering all divisions of Bangladesh. There were several limitations in the study to be mentioned. This surveillance used data collected from sentinel sites, which represents only the most severe cases that had access to hospital care. Therefore, the findings may not represent the true disease burden in the community. Patients were not followed up after discharge; therefore, data on clinical consequences lacked in this study. Moreover, molecular techniques were not performed to confirm the etiology of the infection.

## Conclusion

In summary, the nationwide surveillance showed evidence that infection with enteric hepatitis viruses is common in Bangladesh. The result of this surveillance will shed light on the disease burden in Bangladesh, and the evidence will be an important step towards the development of preventive and control strategies. Public health measures, through control and management of patients, will help to interrupt the transmission cycle, while proper medical management will help to reduce the development of the consequences of chronic liver disease. Moreover, expanded vaccination strategies for acute viral hepatitis and improved sanitation programs may be beneficial as preventive measures in this region, to ultimately reduce the disease burden.
